# Retraction: Involvement of the JNK/FOXO3a/Bim Pathway in Neuronal Apoptosis after Hypoxic–Ischemic Brain Damage in Neonatal Rats

**DOI:** 10.1371/journal.pone.0233069

**Published:** 2020-05-06

**Authors:** The PLOS ONE Editors

After this article [1] was published, concerns were raised about similarities between western blot data reported in Figs 1–4.

Specifically:

- The following panels appear similar: Fig 1A GAPDH, Fig 2E GAPDH, Fig 3A GAPDH, Fig 3G Akt, and Fig 3G GAPDH. Aspect ratios are different between the similar panels in some cases.
- The GAPDH panels appear similar in Fig 2A and 2C, when one is rotated 180 degrees, and Fig 2C GAPDH lanes 2–5 appear similar to Fig 3C FOXO3a lanes 1–4.
- Fig 3C p-FOXO3a and Fig 3E FOXO3a cytoplasm panels appear similar.
- Fig 3C GAPDH (rotated 180 degrees), Fig 3G p-Akt, and Fig 4A GAPDH panels appear similar.
- In Fig 4A, the Bim bands appear similar to the CC3 bands in the middle blot, when rotated 180 degrees.

The above issues have potential implications for the reliability of quantitative data reported in the corresponding Relative Optical Density graphs.

The authors confirmed the duplications and noted that the original data underlying the figures of concern are not available. Without the original raw data, the concerns about data reporting in the published figures cannot be resolved.

The above issues call into question the reliability of the article’s results and conclusions, as a result, the *PLOS ONE* Editors retract this article.

DL, JL, and DM agreed with retraction. The other authors either could not be reached or did not respond directly.
